# Clinical predictors of successful magnetic resonance-guided focused ultrasound (MRgFUS) for uterine leiomyoma

**DOI:** 10.1186/2050-5736-1-15

**Published:** 2013-09-02

**Authors:** Krzysztof R Gorny, Bijan J Borah, Amy L Weaver, Douglas Brown, David A Woodrum, Elizabeth A Stewart, Gina K Hesley

**Affiliations:** 1 Center for Uterine Fibroids, Mayo Clinic and Mayo Clinic College of Medicine, 200 First St, SW, Rochester, MN 55901, USA; 2 Department of Obstetrics and Gynecology, Mayo Clinic and Mayo Clinic College of Medicine, 200 First St, SW, Rochester, MN 55901, USA; 3 Department of Radiology, Mayo Clinic and Mayo Clinic College of Medicine, 200 First St, SW, Rochester, MN 55901, USA; 4 Division of Biomedical Statistics and Informatics, Mayo Clinic and Mayo Clinic College of Medicine, 200 First St, SW, Rochester, MN 55901, USA; 5 Division of Health Care Policy and Research, Mayo Clinic and Mayo Clinic College of Medicine, 200 First St, SW, Rochester MN 55901, USA

**Keywords:** Fibroids, Uterine leiomyoma, Symptomatology, Magnetic resonance-guided focused ultrasound surgery, MRgFUS, Treatment outcomes, Clinical predictors

## Abstract

**Background:**

Magnetic resonance-guided focused ultrasound (MRgFUS) is a relatively new minimally invasive treatment, approved by the US Food and Drug Administration in 2004 for treatments of symptomatic uterine leiomyomas (fibroids). The purpose of this work is to present retrospective cohort analysis of women that underwent commercial MRgFUS treatment between 2005 and 2009 at a single center, to identify baseline patient characteristics that predict successful MRgFUS fibroid treatment. Identifying these clinical predictors of MRgFUS would be helpful to clinicians choosing the optimal patient for this treatment modality.

**Methods:**

One hundred thirty women with symptomatic uterine leiomyomas who underwent MRgFUS were followed up with a mean length of follow up of 17.4 ± 10.3 months. The main outcome measure of the follow-up was to identify patients who required additional fibroid treatment due to continued fibroid symptoms. Additionally, patient medical history and radiological findings obtained prior to MRgFUS were reviewed, and statistical analysis was performed to identify factors associated with reduced risk of having additional fibroid treatment.

**Results:**

Twenty-nine patients (22.3%) underwent additional fibroid treatment due to continued or recurrent fibroid symptoms during the follow up. Cumulative incidence of additional fibroid treatment was 9.7%, 29.3%, and 44.7% at 1, 2, and 3 years following MRgFUS, respectively. In multivariable Cox proportional hazard regression analyses, older age (hazard ratio (HR) 0.54 per 5-year increase in age, 95% confidence interval 0.39 to 0.76, *p* < 0.001), greater number of fibroids (HR 0.19 for more than three vs. one fibroid, 95% CI 0.05 to 0.67, *p* = 0.033), and greater fibroid volume (HR 0.70 per doubling in volume, 95% CI 0.51 to 0.96, *p* = 0.025) were significantly associated with less risk of having additional fibroid treatment.

**Conclusions:**

Older age at treatment and having multiple fibroids with larger volume are associated with a lower risk of additional intervention following MRgFUS treatment for uterine fibroids.

## Introduction

Uterine leiomyomas (fibroids or myoma) are benign clonal tumors that arise from the smooth muscle cells of the myometrium [1]. They are the most common gynecologic neoplasm in women of reproductive age and are clinically noted in up to 25% of all women [2,3]. Studies using both ultrasound and pathologic examination of surgical uterine specimens suggest an overall prevalence of uterine fibroids of over 70% [4-6].

Fibroids can cause a variety of symptoms [7], which can generally be classified into three distinct categories: increased heavy menstrual bleeding, pelvic pressure and pain, termed bulk-related symptoms, and reproductive dysfunction [1].

Historically, hysterectomy has been employed as the major treatment option for uterine fibroids. However, there is an increasing array of less invasive alternatives to hysterectomy for the control of uterine fibroids [3,7]. Magnetic resonance-guided focused ultrasound (MRgFUS) is a relatively new treatment option, which was approved by the US Food and Drug Administration (FDA) in 2004 specifically for treatments of uterine fibroids. This noninvasive thermoablative technique uses phased array ultrasound transducer to focus a beam of ultrasound energy on a target site, causing localized coagulative necrosis. The transducer is integrated with a magnetic resonance imaging (MRI) scanner. MRI is used for anatomical treatment planning, ultrasound beam guidance and near real-time thermal feedback, and ablation assessment upon completion of treatment.

There is accumulating evidence on the efficacy and safety of MRgFUS in the treatment of symptomatic uterine leiomyomas [8]. However, little is known which fibroid patients are best candidates for this treatment modality. Recently published retrospective analysis of 81 patients [9] indicated that older women with fibroids presenting as hypointense in T2-weighted MR images are more likely to benefit from MRgFUS. In this work, we present similar analysis based on 130 patients treated between March 2005 and December 2009 at our institution. Description of this patient cohort, details of the treatments, and their 12-month outcome were presented elsewhere [10-12]. Our aim in this work is to determine which patient characteristics available to the physician in the screening process can predict successful MRgFUS treatment (defined as lack of additional intervention due to fibroid-related symptoms). The analysis is based on pretreatment patient screening, which includes MRI findings and review of patient medical history and presenting symptoms.

## Materials and methods

This retrospective cohort analysis was conducted at Mayo Clinic, Rochester, Minnesota. Women with symptomatic uterine fibroids treated with MRgFUS between March 2005 and December 2009 were included in the study. Patients who denied the use of their data for research purposes were excluded. The study was approved by the Mayo Clinic Institutional Review Board (IRB).

### MRgFUS treatments

Treatments were performed using the ExAblate 2000 focused ultrasound device (InSightec, Haifa, Israel) integrated with MRI scanner (Signa Excite, GE Healthcare, Milwaukee, Wisconsin, USA). Details of the MRgFUS treatments have been published elsewhere [10-12]. All patients underwent a pretreatment MRI with gadolinium contrast. Patients were treated on an outpatient basis. Intravenous conscious sedation, using combination of midazolam hydrochloride and fentanyl citrate (typical dose of 0.5 mg midazolam and 25 μg fentanyl), was used during the course of the procedure to maintain patient comfort and allow communication with the treating physician [13]. At the end of the procedure, MR images with gadolinium contrast were acquired to assess the treatment and measure the non-perfused (i.e., ablated) volume (NPV), which previous studies have shown to correlate with the volume of coagulative necrosis caused by treatment [13].

### Fibroid classification and volume measurements

Methods described previously by Gorny et al. [12] were used in image analysis and fibroid volume measurements. The measurements were performed in consensus between two radiologists and a medical physicist experienced in MRI and MRgFUS treatments (all with at least 7 years of experience). Fibroids were categorized according to their T2 signal intensity in the MRI screening: ‘dark with minimal heterogeneity’ (signal intensity lower than that of myometrium, less than 25% heterogeneous), ‘dark with substantial heterogeneity’ (signal intensity lower than that of myometrium, more than 25% heterogeneous), ‘isointense’ (signal intensity equal to that of myometrium), and ‘bright’ (signal intensity greater than that of myometrium). The volumetric measurements were performed using Vitrea® 2.2 (ver. 3.0, Vital Images, Inc., Minnetonka, MN, USA) segmentation software. T2-weighted treatment-planning images were used to measure fibroid volumes. T1-weighted images, with gadolinium contrast, acquired upon completion of the treatment, were used to measure NPV defined as the sum of all non-perfused (ablated) fibroid tissues. For each patient, the following quantities were recorded: the cumulative volume of all fibroids within the uterus (total fibroid load), the volume of the dominant (largest) fibroid, and the ratio of NPV to the total fibroid load (NPV ratio).

Following the focused ultrasound procedure, patients were interviewed by phone after 3 and 6 months and yearly thereafter to assess symptom relief and additional procedures undertaken for relief of fibroid-related symptoms. From the beginning of 2007, patients were additionally asked to complete the Uterine Fibroid Symptom Quality-of-Life [14] questionnaire at baseline, and 6 and 12 months of follow up.

### Patient follow up

For each patient, an extensive review of ambulatory and inpatient medical records at our institution was conducted. Information regarding patient demographics, disease history and symptoms, and NPV and NPV ratios was abstracted. The primary outcome assessed was whether or not the patient had an additional fibroid procedure (e.g., hysterectomy, myomectomy, MRgFUS) during the follow up. The treatment was considered successful if the patient did not have an additional procedure for fibroid-related symptoms during the follow-up period. Additional procedures due to continued or recurrent fibroid symptoms were limited to subsequent hysterectomy, myomectomy, uterine artery embolization (UAE), endometrial ablation, or retreatment with MRGFUS. Hormonal gonadotropin-releasing hormone (GnRH) agonist therapy during the follow-up period (such as Lupron) was not considered as an additional procedure.

### Statistical analysis

Time-to-event methodologies were used to evaluate having additional treatment due to continued fibroid symptoms or recurrence of fibroid symptoms, taking into account the varying duration of follow up. Duration of the follow up was calculated from the time of the initial MRgFUS procedure to the date of the subsequent additional procedure for women failing the treatment. For women undergoing an additional procedure for reasons other than symptom recurrence, their follow up was censored at the date of that additional procedure. For patients who did not undergo additional procedures during the follow-up period, the follow up was censored at the date of last contact or at the onset of menopause. The Kaplan-Meier method was used to estimate the cumulative incidence of additional procedures [15]. Cox proportional hazard models were fit to evaluate factors associated with the need for additional procedure [16]. Univariate models were fit to identify variables with a *p* value of less than 0.40 as candidate predictors of having an additional treatment. The candidate predictors were then considered in a multivariate Cox proportional hazard analysis, in which a parsimonious model was identified using stepwise and backward variable selection methods. Associations were summarized using the hazard ratio (HR) and corresponding 95% confidence intervals (CI). All calculated *p* values were two-sided, and *p* values less than 0.05 were considered statistically significant.

## Results

Between March 2005 and December 2009, 144 women completed MRgFUS treatment at our institution. Fourteen patients denied the use of their data for research purposes prior to treatment; therefore, our study cohort consists of 130 women. Descriptive statistics of the treatments and their immediate outcomes are provided elsewhere [10-12].

Characteristics and history of the cohort are presented in Tables 1 and 2. The mean length of follow up in our cohort was 17.4 ± 10.3 months. Over this period, 29 patients received additional treatment due to continued or recurrent fibroid symptoms. The cumulative incidences of additional fibroid treatment were 9.7% (95% CI 4.1 to 15.0%; number still at risk 94), 29.3% (95% CI 18.0 to 39.1%; number still at risk 35), and 44.7% (95% CI 26.4 to 58.6%; number still at risk 12), 1, 2, and 3 years following MRgFUS treatment, respectively. ‘Number still at risk’ represents the number of patients with a duration of follow up more than *t*, at *t* years following the initial MRgFUS. The mean interval of additional treatment was 16.7 ± 10.1 months, the earliest occurring 6.2 months following MRgFUS, and the latest at 49.6 months. Among the patients who underwent additional treatments, 19 had hysterectomy, 6 had myomectomy, 2 had uterine artery embolization, 1 had endometrial ablation, and 1 had retreatment with MRgFUS. Furthermore, there were three patients who underwent additional treatment for reasons other than fibroid-related symptoms: one patient underwent a hysterectomy because her gynecologist was not able to successfully perform a Papanicolaou test, one woman underwent a myomectomy during surgery for a pancreatic tumor, and one patient underwent myomectomy because of infertility issues.

**Table 1:** **Characteristics of the cohort (*n*** **= 130)**

**Characteristic**	**Mean ± SD**
Patient	
Age at treatment (years)	45.1 ± 5.5
BMI (kg/m^2^) (*n* = 95)	
<25	52 (54.7)
25 to 29	26 (27.4)
≥30	17 (17.9)
Race (*n* = 88)	
Caucasian	77 (87.5)
Other	11 (12.5)
Smoking history (*n* = 123)	
No	82 (66.6)
Yes	41 (33.3)
Gravidity (*n* = 129)	
Nulligravid	59 (45.7)
Multigravid	70 (54.3)
Parity (n = 129)	
Nulliparous	70 (54.3)
Multiparous	59 (45.7)
Menopausal status (*n* = 129)	
Premenopausal	110 (92.4)
Perimenopausal	8 (6.7)
Postmenopausal	1 (0.8)
Prior fibroid treatment	
None	96 (73.8)
Medical	22 (16.9)
Myomectomy	2 (1.5)
Endometrial ablation	2 (1.5)
MRgFUS	8 (6.2)
Symptom	
Age at first diagnosis (years) (*n* = 104)	41.5 ± 6.2
Duration of dominant symptom (months) (*n* = 98)	20.6 ± 15.7
Presenting symptoms (*n* = 124)	
Bleeding + Bulk	39 (31.0)
Bleeding only	32 (25.4)
Bulk only	35 (27.8)
Pain (±Bleeding or Bulk)	20 (15.9)
Baseline symptom severity score (*n* = 66)	59.1 ± 18.0
Fibroid	
Fibroid load (cm^3^)	350 ± 307
Volume dominant fibroid (cm^3^)	305 ± 292
Number of fibroids	
1	50 (38.5)
2 or 3	39 (30.0)
>3	41 (31.5)
Fibroid location	
Submucosal intramural	14 (10.8)
Intramural	86 (66.2)
Subserosal	22 (16.9)
Transmural^a^	8 (6.2)
T_2_ signal intensity dark, minimal heterogeneity	71 (54.6)
Minimal heterogeneity	
Minimal heterogeneity	71 (54.6)
Substantial heterogeneity	44 (33.8)
Isointense	6 (4.6)
Bright	9 (6.9)

**Table 2:** **History of the cohort (*n*** **= 130)**

**History**	***n *** **(%)**
Medical	
Abnormal pap smear	30 (23.1)
Adenomyosis	4 (3.1)
Anemia	50 (38.5)
Asthma	13 (10.0)
Depression	29 (22.3)
Endometriosis	9 (6.9)
Hypertension	9 (6.9)
Infertility	11 (8.5)
Migraine	28 (21.5)
Sexually transmitted disease	9 (6.9)
Thyroid disease	20 (15.4)
Medication	
Analgesics	28 (21.5)
Antihypertensive medication	12 (9.2)
Iron suppletion	33 (25.4)
GnRH agonist	7 (5.4)
Oral contraceptives, psychofarmaca	27 (20.8)
	18 (13.8)
Thyroid hormone	18 (13.8)
Surgical	
Appendectomy	12 (9.2)
Caesarian section	5 (3.8)
Dilatation and curettage	18 (13.8)
Laparoscopy	10 (7.7)
Laparotomy	9 (6.9)
Pelvic floor reconstruction	1 (0.8)
Tubal ligation	16 (12.3)
Surgery for cervical dysplasia	15 (11.5)

Cell counts may not sum to or exceed 130 because of missing data or multiple conditions that apply; ^a^Term ‘Transmural’ refers to a fibroid that is predominately intramural but occupies full thickness of the myometrium, extending from the endometrium to the serosa.

**Figure 1:**
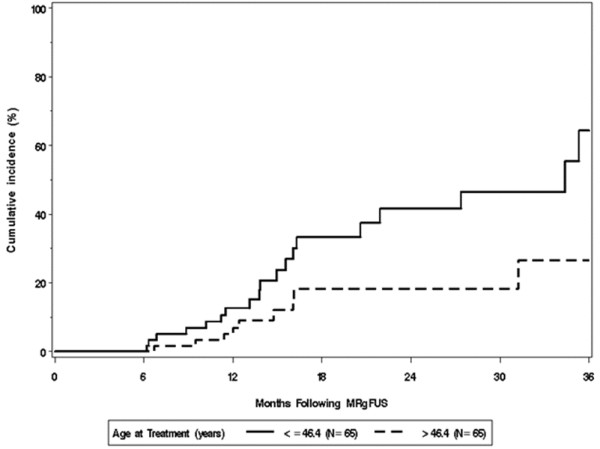
Kaplan-Meier estimates of cumulative incidence of additional fibroid treatment by age subgroups.

Table 3 summarizes patient and fibroid characteristics that were evaluated using univariate Cox models for an association with subsequent treatment for fibroids. As illustrated in Figure 1, women who were initially treated at an older age were significantly less likely to have subsequent treatment; the estimated hazard or risk ratio was 0.63 per 5-year increase in age (*p* = 0.003), implying that an increase in age of 5 years is associated with 37% less likelihood of having an additional procedure. Women with a history of pelvic or uterine surgery were 2.3 times more likely to have subsequent treatment for fibroids compared to women without a prior surgery (*p* = 0.042). In addition, (*n* = 7) women who previously used GnRH agonist (e.g., Lupron) therapy were 3.9 times more likely to have subsequent treatment for fibroids compared to (*n* = 123) women who never used GnRH agonist (*p* = 0.013). The median NPV values were not significantly between the two groups (115.7 (*n* = 7) vs. 126 (*n* = 123), Wilcoxon rank sum test *p* = 0.31).

**Table 3:** Patient and fibroid characteristics evaluated using univariate analysis

**Characteristic**	**HR (95% CI)**^**a**^	**Univariate Cox models *p *** **value**
Age at treatment (years)	0.63 (0.46 to 0.86)	0.003
BMI (kg/m^2^)	0.90 (0.64 to 1.28)	0.57
Ever pregnant (yes vs. no)	0.72 (0.34 to 1.52)	0.39
Ever live birth (yes vs. no)	0.80 (0.38 to 1.71)	0.57
Smoking history (yes vs. no)	0.45 (0.18 to 1.12)	0.09
Symptom severity score (SSS)^b^	1.18 (0.87 to 1.59)	0.29
Duration of dominant symptom (months)	0.95 (0.79 to 1.15)	0.60
Pelvic pressure (yes vs. no)	0.42 (0.20 to 0.89)	0.023
Back pain (yes vs. no)	0.53 (0.13 to 2.26)	0.39
Presenting symptoms		0.94
Bleeding only	Referent	
Bulk only	0.78 (0.27 to 2.25)	
Bleeding and bulk	0.77 (0.30 to 1.96)	
Pain (with or without bleeding or bulk)	0.76 (0.23 to 2.51)	
Past medical history		
Anemia (yes vs. no)	0.50 (0.22 to 1.13)	0.10
Hypertension (yes vs. no)	1.95 (0.46 to 8.36)	0.37
History of asthma (yes vs. no)	^c^	0.10
History of migraines (yes vs. no)	2.00 (0.92 to 4.35)	0.08
History of pelvic or uterine surgery (yes vs. no)	2.29 (1.03 to 5.08)	0.042
Thyroid disease (yes vs. no)	1.56 (0.63 to 3.84)	0.34
Prior GnRH agonist therapy	3.92 (1.33 to 11.51)	0.013
Prior usage of oral contraceptives (yes vs. no)	2.02 (0.84 to 4.85)	0.11
Prior iron supplementation (yes vs. no)	0.63 (0.26 to 1.56)	0.32
Prior thyroid hormone therapy (yes vs. no)	1.56 (0.63 to 3.84)	0.34
Fibroid characteristic		
Prior treatment (yes vs. no)	1.58 (0.71 to 3.51)	0.26
Fibroid type		0.52
Submucosal	1.36 (0.51 to 3.65)	
Subserosal	0.4 (0.09 to 1.72)	
Transmural	0.78 (0.18 to 3.35)	
Intramural	Referent	
Number of fibroids		0.029
1	Referent	
2 to 3	0.65 (0.28 to 1.52)	
>3	0.19 (0.06 to 0.66)	
Volume of largest fibroid (cm^3^)	0.80 (0.61 to 1.06)	0.11
Total fibroid volume (cm^3^)	0.70 (0.52 to 0.95)	0.022
T2 signal intensity		
“Dark with min. heterogeneity” vs. all others	0.73 (0.35 to 153)	0.40
All dark vs. “isointense” and “bright”	0.85 (0.29 to 2.45)	0.76

Patient characteristics were evaluated for an association with subsequent treatment (*p* values based on the Wald test within the Cox model); ^a^HR, hazard ratio estimated from univariate Cox regression models. HR per 5-year increase in age, 5-unit increase in BMI, 10-unit increase in SSS score, 6-month increase in the duration of symptoms, doubling in volume of largest fibroid, and doubling in total fibroid volume; ^b^10-point Symptom Severity Scale of the Uterine Fibroid Symptom Quality-of-Life (UFSQOL) questionnaire; ^c^Unable to estimate the HR for a history of asthma since none of the patients with a history of asthma have had subsequent fibroid treatment.

**Figure 2:**
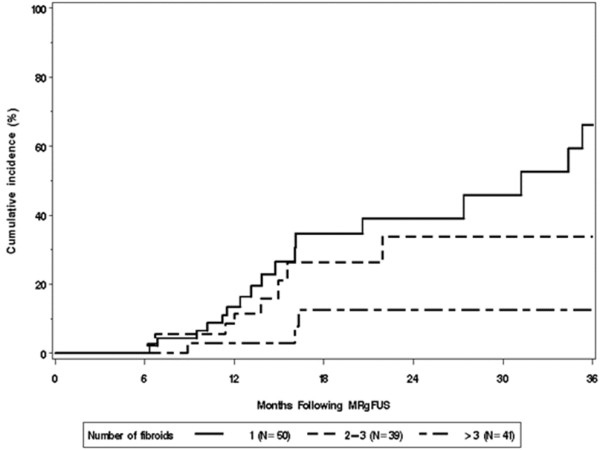
**Kaplan-Meier estimates of cumulative incidence of additional fibroid treatment by the number of fibroids.** Patients with either two to three or more than three fibroids were less likely to have subsequent treatment compared to those with a single fibroid (*p* = 0.029).

**Figure 3:**
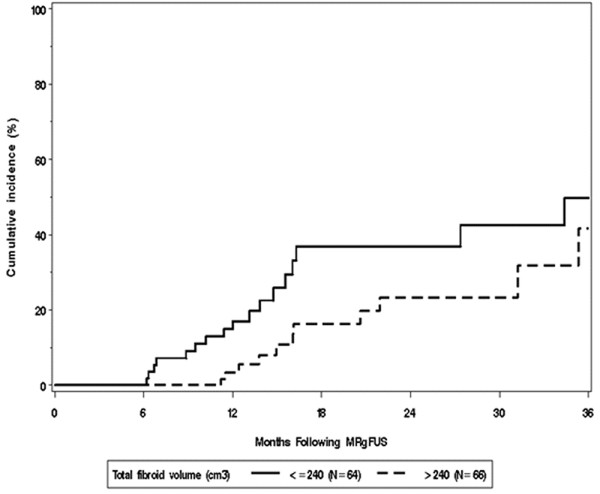
Kaplan-Meier estimates of cumulative incidence of additional fibroid treatment by total fibroid volume subgroups.

Patients with either two to three or more than three fibroids were less likely to have subsequent treatment compared to those with a single fibroid (Figure 2, *p* = 0.029). As illustrated in Figure 3, women with a greater total fibroid volume were less likely to have subsequent treatment; the estimated hazard ratio was 0.70 per a doubling in fibroid volume (*p* = 0.022).

The results of the multivariable analysis are summarized in Table 4. In the multivariable Cox model, older age (HR = 0.54 per 5-year increase in age, 95% CI 0.39 to 0.76, *p* < 0.001), greater number of fibroids (HR = 0.19 for more than three vs. one fibroid, 95% CI 0.05 to 0.67, *p* = 0.033), and greater fibroid volume (HR = 0.70 per doubling in volume, 95% CI 0.51 to 0.96, *p* = 0.025) were significantly associated with less risk of having additional fibroid treatment.

**Table 4:** Results of multivariate analysis

**Characteristic**	**HR (95% CI)**^**a**^	***P *** **Value**
Age at treatment (years)	0.54 (0.39 to 0.76)	<0.001
Number of fibroids		0.033
1	Referent	
2 to 3	0.89 (0.37 to 2.14)	
>3	0.19 (0.05 to 0.67)	
Total fibroid volume (cm^3^)	0.70 (0.51 to 0.96)	0.025

^a^HR, hazard ratio per 5-year increase in age and doubling in total fibroid volume, respectively.

## Discussion

MRgFUS is a minimally invasive procedure and unique such that it is a noninvasive treatment which is administered transabdominally. Appropriate patient selection is essential to maximize the treatment's efficacy, and by identifying factors that influence treatment outcome, the profile of an ideal candidate for MRgFUS can be developed [17]. Knowledge of recurrence rates and prognostic variables is useful to clinicians counseling patients and choosing the optimal treatment.

Following MRgFUS treatment, fibroid-related symptoms are typically expected to improve within several months. For this reason, all the patients had the opportunity of at least 6 months of follow up as the last MRgFUS treatment was in December 2009, and the follow up was assessed through July 2010. However, nine patients have less than 6 months of follow up either because they were lost to follow up (*n* = 8) or had a myomectomy for non-fibroid-related issues (*n* = 1). These women have all been retained in the analysis and were appropriately censored in the time-to-event analyses.

Every uterus-sparing treatment modality for leiomyomas has a substantial risk of clinical failure due to the possibility of leiomyoma recurrence. Several groups reported on recurrence rates and additional treatments associated with uterine-sparing fibroid therapies such as myomectomy [18-20] and UAE [21-23]. Cumulative risk of recurrence 5 years following abdominal myomectomy was reported as 62% (9% additional treatments, report based on 145 patients) [20]. For laparoscopic myomectomy, reported cumulative recurrence risks were 31.7% in 3 years and 51.5% in 5 years (12% additional treatments, study based on 114 patients) [19]. In a prospective long-term follow-up study of 200 patients after UAE, Spies et al. [23] found rate of recurrence or symptom control failure of 25% (19.7% additional treatments) 5 years after embolization. In our cohort, cumulative rates of additional fibroid treatment in 1, 2, and 3 years, were respectively 9.7%, 29.3%, and 44.7%, which are within the upper range of data reported for myomectomy and UAE. Our patient population could arguably be at a higher than average risk of needing additional intervention since a quarter of the women had already had one fibroid intervention which failed to control their symptoms. Additionally, this series reports on the earliest non-study experience with a novel technique with no predicate learning curve and known suboptimal treatments in many cases.

In agreement with the recent published report on clinical predictors of treatment success [9], we find that MRgFUS treatment at a younger age is associated with increased risk of needing additional treatment. One simple explanation for this finding is that the time duration to the onset of menopause for younger women is longer leaving them at a higher risk of fibroid recurrence or a clinical treatment failure. It could also be argued that the severity of the fibroid disease (measured by the severity of symptoms) is increased in women with an earlier disease onset, which could lead to higher recurrence rates [23].

Higher risk of additional intervention was found in women with single vs. multiple fibroids and in women with a lower fibroid volume. There have been somewhat conflicting reports of similar analyses on the predictors of abdominal myomectomy and UAE. While our result supports the findings of Stewart et al. [24], it is in contrast with those of Hanafi [20] who reported the cumulative recurrence 5 years after abdominal myomectomy to be significantly lower in patients with single leiomyomata than in those with multiple leiomyomata. Similarly, Spies et al. [23] also associated larger fibroid volume with higher risk of further intervention. The size and location of uterine fibroids are related to different karotypic abnormalities representing differences in gene expression [24-27]. Therefore, these conflicting results could be associated with different biologic expressions of the fibroid disease and varied response to treatments and different patient populations. Future studies are necessary to correlate cytogenetic studies with clinical outcomes. Lastly, our result may perhaps reflect some difficulty of MRgFUS to adequately treat the entire volume of a single dominant fibroid vs. smaller fibroids.

Prior studies have shown that fibroids which are hypointense on screening T2-weighted MRI respond optimally to MRgFUS with maximized non-perfused volume (NPV) and highest shrinkage following treatment [28], and lower risk of additional fibroid intervention in long-term follow up [9]. In our cohort, data regarding T2 signal intensity were analyzed from the perspective of ‘dark with minimal heterogeneity’ vs. all others, and ‘all dark’ (regardless of the degree of their heterogeneity) vs. all others. Regardless of the approach, no statistically significant association between the fibroid T2 appearance and either of these key outcomes was found (see Table 3). One likely explanation for this is that a relatively small population in ‘isointense’ and ‘bright’ fibroid groups in our patient cohort (11.6% of all fibroids) inhibits in-depth statistical analysis.”

The role of MRgFUS is similar to the role of other uterine-sparing procedures, which all have risks of recurrence and further interventions. The finding that younger age at treatment is associated with increased risk of needing further intervention may be viewed as disappointing to younger women. However, in the light of the recently reported high rate of ongoing and delivered pregnancies, post-MRgFUS [29], this therapeutic modality should be considered as a reasonable and safe treatment option to those young women who wish to conceive.

It is reassuring that presenting symptomatology did not influence treatment outcome. Moreover, concurrent diagnoses such as adenomyosis or endometriosis were not significant factors, but we lack statistical power to make meaningful statistical conclusion since few of our patients had these conditions at the time of treatment.

The limitations of the study include the retrospective study design and significant prevalence of women of Caucasian descent. Also, defining successful treatment in terms of lack of additional interventions is somewhat limited since it potentially excludes patients who had either recurrence of symptoms, or insufficient symptom relief but did not seek further medical treatment.

In conclusion, we find that older women, with several fibroids and higher fibroid volume are more likely to benefit from the MRgFUS treatment. Additionally, the incidence of additional treatments post MRgFUS for fibroid-related symptoms (during the follow up) is comparable with those published for other minimally invasive uterine-sparing treatments (e.g., UAE).

## Competing interests

KG, GH, DW, and ES received support through RC1HD063312 and R01HD060503 from the Eunice Kennedy Shriver National Institute of Child Health and Human Development of the NIH. KG, GH, and ES received salary support for a separate clinical trial agreement between Mayo Clinic and InSightec (Haifa, Israel). ES is a consultant to Gynesonics and Abbott and serves on the Scientific Advisory Board for the Bayer HealthCare Scientific Committee.

## Authors’ contributions

KRG, DB, DAW, EAS, and GKH performed data collection, data analysis, and contributed to the design and writing of the manuscript. BJB and ALW performed statistical analysis of the data and contributed to the design and writing of the manuscript. All authors read and approved the final manuscript.
